# Modified Gut Anastomotic Technique in Type III and Type IV Jejunoileal Atresias

**DOI:** 10.21699/jns.v5i4.403

**Published:** 2016-10-10

**Authors:** Kamal Nain Rattan, Deepak Kumar Garg

**Affiliations:** Department of Pediatric Surgery, Pt B. D. Sharma Pgims Rohtak Haryana, India

**Keywords:** Intestinal atresia, Jejunoileal atresia, Anastomosis, Modification

## Abstract

Background: Type III and IV jejunoileal atresias are associated with loss of significant length of the gut and can lead to short gut syndrome if further resection of proximal dilated gut is done. We modified the anastomotic technique so that proximal dilated segment of the gut is not resected as to prevent short gut syndrome.

Material and Methods: Medical Record of patients of Type III and IV jejuno-ileal atresias managed with modified anastomotic technique in our center during 5-years was reviewed.

Results: Fifteen patients were managed with our modified technique. There were no anastomotic leak observed and there was 6% mortality seen in our modified technique.

Conclusion: We found less mortality and morbidity in our technique compared to recommended techniques described in literature.

## INTRODUCTION

Intestinal atresia is one of the commonest causes of intestinal obstruction in neonates, which occurs from one in 400 to one in 5000 newborns.[1] Improvement in anesthesiology and perioperative care leads to better survival. The survival rate of jejunoileal atresia in the western world is above 89%.[2] Indian study by Bhagwat et al shows 68% survival.[2] Grosfeld et al modified Louw's original classification into four classes of intestinal atresia, which is currently the most commonly used classification scheme has both prognostic and therapeutic implications.[3] 

In 1911, Fockens reported the first successful surgical repair of a patient with small intestinal atresia.[4,5] However, the mortality associated with surgical correction of this condition remained high for many years, even in the best pediatric surgical centers.[5]

## MATERIALS AND METHODS

It is a case series of 15 patients who were managed with modified anastomotic technique. The neonates were admitted with failure to pass meconium, abdominal distension and bilious vomiting. Two neonates had jaundice. The majority of neonates presented during 2nd to 5th day. Gestational age varied from 32 to 40 weeks and birth weight varied from 1.1 to 3.4 kg. 

Patients were kept nil-per-oral and resuscitated with the nasogastric tube for gut decompression and judicious IV fluids and antibiotics. After correction of dehydration and optimization of serum electrolyte and renal function, they were investigated with xray abdomen. 

Modified operative technique: All the patients were operated through a right transverse supraumbilical incision with supine position. Abdomen was explored, for a type of atresia, saline was injected in distal collapsed segment to rule out multiple atresias and associated anomalies. A 2 cm incision was made which was perpendicular to gut lumen at the summit of atretic proximal dilated end and end to end anastomosis was done with horizontal mattress suture with small bite distance on the distal collapsed segment and larger bite on the dilated segment with 5-0 silk suture as described in (Fig. 1,2). Defect in the mesentery was closed.

**Figure:**
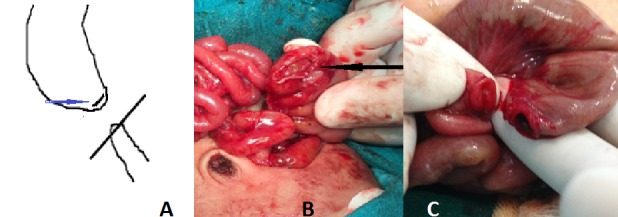
Figure:1 A) Line diagram showing the level of incision which was perpendicular to the gut and involved summit of the distal end of proximal dilated segment. (solid blue arrow pointing the incision line in proximal segment). B) Proximal segment after incision.(shown by black arrow). C) Proximal and distal end after incision.

**Figure:**
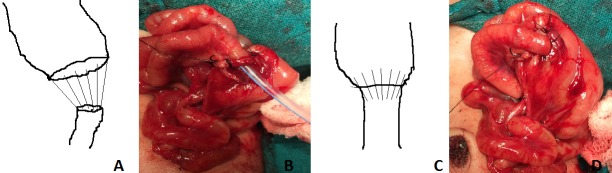
Figure 2: A) line diagram showing that posterior layer was closed with horizontal mattress full-thickness bites (from inside-out in the proximal gut than out-inside in distal gut inside-out in the distal gut than slightly wider bite distance than distal gut out-inside) and the distance between the bites were more in proximal dilated gut than the distal gut segment. Anterior layer closed with the seromuscular sutures in a similar manner to the posterior layer. B) Showing picture after posterior layer of anastomotic closure. C)Line diagram showing the gut after the gut anastomosis (anastomotic suture placement as more gap between suture in dilated proximal part). D) Showing picture after completion of anastomosis.

Postoperatively, nasogastric tube drainage progressively decreased and removed on average after 6.2 day. After passage of stool, breast feeding was started. Average hospital stay was 12-14 days.

## RESULTS

Out of 15 patients, one patient expired on day 19th due to sepsis which was due to aspiration pneumonitis. One patients developed wound discharge which was managed with dressing and antibiotics. There was no anastomotic leak observed in these patients. Another child presented with wound dehiscence and discharge in follow-up was readmitted for wound care but patients had no anastomotic leak as shown in Table 1.

**Figure:**
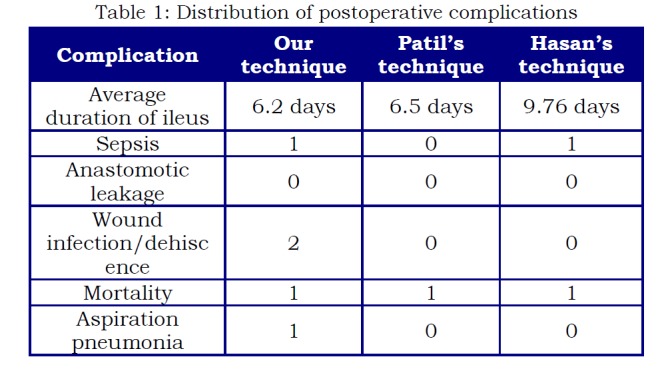
Table 1: Distribution of postoperative complications

## DISCUSSION

In 1894 Wanisschek attempted the first resection anastomosis for intestinal atresia unsuccessfully. Since then, many procedures have been attempted to establish bowel continuity, avoid anastomotic complications and prevent short bowel syndrome. The final decision on the most appropriate procedure depends on the type of obstruction, associated malrotation meconium ileus, meconium peritonitis and extent of the atretic segments.[6] 

Many anastomotic techniques have been devised for intestinal atresia. The procedures can be classified into two types: 1) widening of a caliber of the diminutive distal bowel and 2) reducing caliber of the large proximal bowel. End to back, end to side, and end to end oblique anastomosis that is described belongs to the first type, and tapering enteroplasty followed by end to end anastomosis into the second type.[6]

Denis Browne's end to back technique involves resection of a significant length of the proximal atretic segment with an antimesenteric cut in the distal segment to match the lumen of the proximal segment. In this technique, there can be angulation at the suture line. In near non-compressible systems, when fluid flows around a corner, an additional hydrostatic pressure is exerted at the bend: this will act as a shearing force at the anastomosis site (bernoulli's hydrodynamic principle). [2]

In the Patil's end to end linear anastomotic technique, the tip of the distal segment and disc matching the size of the distal atretic segment were excised from the apex of the dilated proximal atretic segment and a single layer anastomosis was done using 5-0 interrupted vicryl sutures. Several plicating sutures were used for reducing the proximal gut lumen by inverting the bowel. In the Patil's technique, plication requires passage of sutures through the proximal dilated segment which seems unnecessary, as without plication there was no increase in leak rate was found in our series. [2] 

In Hasan's concavoconvex oblique anastomotic technique, proximal bowel cut in concave fashion after 5-10 cm resection and distal bowel in convex fashion and performed in an intermittent manner with vicryl 5-0. But Hasan's technique involve resection of proximal segment and anastomosis is oblique leads to a shearing force which predisposes for the higher rate of anastomotic leak.[6] 

In our technique as described above, we didn't resect the proximal dilated segment and doing end to end anastomosis without any plication of proximal dilated segment to decreased the lumen diameter and we have not found any angulation and anastomotic leak. It is less time consuming simple anastomotic technique with no resection of gut or cheatle maneuver or plication. 

As our approach save the small intestinal length as these child have already compromised length and sometime require multiple surgeries which ultimately leads to parenteral nutrition dependency and short bowel syndrome and there is no axial deviation seen as seen with most of the other types. Dalla et al in there study concluded that ultra-short bowel syndrome was the most common cause of morbidity and mortality in jejunoileal atresias [7,8]. 

Lumen discrepancies present during anastomosis between proximal dilated lumen and distal constricted lumen. It has been found that there is smooth muscle hypertrophy, hyperplasia and a paucity of ganglion cells in the proximal bowel. But the smooth muscle changes are secondary to obstruction and will revert back to normal as distal bowel regain its function.[2] although our series include 15 patients, but there was no anastomotic leak and lesser mortality is observed (6%) and it is easy and less time consuming.

## CONCLUSION

There is no need to resect or plication of the dilated segment in these patient as there is no benefit in terms of morbidity and mortality and there is lesser morbidity and mortality seen in our new technique, so we recommend this method of bowel anastomosis in cases of type III and IV jejunoileal atresia (where further resection may lead to short bowel syndrome) as a technical advancement.

## Footnotes

**Source of Support:** None

**Conflict of Interest:** None
